# Differentiation Treatment Applied to Lung Cancer Model Reduces Pathogenic Traits in Vitro

**DOI:** 10.1002/adbi.202500371

**Published:** 2025-11-29

**Authors:** Alice Grossi, Paola Fulghieri, Abdurakhmon Aduvaliev, Karen Soffiantini, Irene Oldrati, Margherita Cavallo, Marco Biggiogera, Giorgia Pellavio, Umberto Laforenza, Monica Savio, Virginie Sottile

**Affiliations:** ^1^ Department of Molecular Medicine University of Pavia Pavia Italy; ^2^ Department of Biology and Biotechnology University of Pavia Pavia Italy; ^3^ Centre for Health Technologies (CHT) University of Pavia Pavia Italy; ^4^ UOC Bioscaffolds and transplantation Fondazione IRCCS Policlinico San Matteo Pavia Italy

**Keywords:** differentiation, inhibition, lung adenocarcinoma model, migration, stem cell markers

## Abstract

Non‐small cell lung cancer (NSCLC) relapse after therapy is linked to the high aggressiveness, chemoresistance and metastatic potential of tumor cells due, in part, to the presence of cancer stem cells (CSCs). Pro‐differentiation approaches have shown promising results for leukemia and in some solid cancer models, offering a possibility to enhance current anti‐cancer therapies. Here, the human NSCLC line A549 is exposed to a serum‐containing medium supplemented with pro‐differentiation factors (DM), and effects on the cells’ proliferation, migration and adhesion properties are assessed in vitro, alongside CSC marker expression analyzed after treatment in 2D or 3D culture conditions. A549 cells exposed to DM exhibited notable morphological changes, with significant increase in cellular footprint and vesicle accumulation. These phenotypic alterations coincided with significant inhibition of proliferation and migration, whereas adhesion properties increased, similar to alkaline phosphatase activity. DM treatment of A549 cells also caused a significant reduction in clonogenic ability by two thirds, as well as halving anchorage‐independent colony formation and spheroid growth, alongside a reduced expression of stemness markers SOX2, NANOG, CD44 and ABCG2, and of ALDH activity and aquaporin function. These results indicate decreased pathogenic features of NSCLC cells after DM exposure, suggesting that pro‐differentiation treatment may represent a valuable option for further preclinical testing.

## Graphical abstract

Non‐small cell lung cancer cells (A549) treated with a differentiation medium showed strong reduction in proliferation, migration, clonogenicity, spheroid formation and stemness markers, with enhanced adhesion properties and alveolar features, all pointing to a notably less aggressive phenotype in support of differentiation therapy as a promising approach for lung cancer.

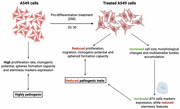

## 1 Introduction

Lung cancer is the second most common cancer in men (after prostate cancer) and the third most common cancer in women (after breast and colorectal cancer) [1], thus representing the first most lethal cancer worldwide in both sexes [2]. The main causes for its high mortality are the aggressiveness of the disease and its late diagnosis, due to scarcity of symptoms at the early stages of development [3].

Among lung cancers, Non‐Small Cell Lung Cancer (NSCLC) represents 85% of all cases of lung tumors [4, 5] and includes a variety of neoplastic types, among which lung adenocarcinoma, a malignant neoplasm that affects the epithelial lining portions of the secretory mucosae. Several therapeutic strategies aimed at treating patients have been developed, according to the tumor's clinical stage and inter‐individual factors, including the traditional approach by surgical removal, radiation therapy to stimulate the necrotic process in tumor cells, and chemotherapy through the administration of one or a combination of cytotoxic drugs inducing the death of neoplastic cells. Another possible treatment strategy is targeted therapy toward proteins involved in the modulation of tumorigenesis, growth, division, and survival of tumor cells. Finally, immunotherapy can be applied to stimulate the host's immune system with the aim of targeting tumor cells [6, 7].

However, the incidence and occurrence of relapses in NSCLC after therapy are steadily increasing due to the high aggressiveness, chemoresistance and metastatic potential induced, in part, by the presence within the tumor mass of cancer stem cells (CSCs), a small subpopulation present in the tumor with characteristics of self‐renewal and differentiation comparable to those of canonical stem cells [7]. In CSCs, the expression of genes regulating growth and cellular division trigger chromatin reshaping and dysregulated gene expression [8, 9] that allow them to hijack homeostatic programs and to dedifferentiated malignant properties such as invasion and migration, lineage plasticity and therapy resistance [10, 11, 12]. Among stemness markers expressed in CSCs are SOX2, CD44, and OCT4, as well as proteins such as ATP‐binding cassette (ABC) transporters, aldehyde dehydrogenase, and epithelial cell adhesion molecule (EpCAM) [13]. With its stemness features, this cell population is considered potentially responsible for the onset of new tumors according to the hierarchical and the dynamic models of carcinogenesis [14, 15].

Several differentiation‐based experimental approaches have recently been proposed with the aim to force differentiation of CSCs into more committed and less aggressive cellular types [16, 17, 18]. The initial success of differentiation therapy as a complement to chemotherapy was the treatment of acute promyelocytic leukemia (APL) [19, 20], and some promising preclinical results were more recently reported in a pre‐clinical breast cancer model [21], offering the possibility of enhancing the effects of current antiproliferative therapies. Of note, Ishay‐Ronen and colleagues pioneered a transdifferentiation treatment to convert mammary carcinoma cells into post‐mitotic adipocytes, with marked success both in vitro and in an animal model [16, 21]. These results open the possibility that selected pro‐differentiation treatments may decrease cancer cell pathogenicity.

Starting from these premises, the present study set out to examine whether this strategy could be applicable and adapted to a human lung cancer model. The NSCLC A549 lung adenocarcinoma cell line was exposed to a similar treatment with a medium containing serum and pro‐differentiation factors (defined as DM), and its response was compared to that observed in standard growth medium used as a control (defined as SM). Effects on the proliferative, migratory and adhesion properties of the treated cells were assessed in vitro, alongside the analysis of clonogenicity and stemness marker expression after treatment in both 2D or 3D culture conditions to evaluate the effect of DM on lung cancer cells.

## 2 Materials and Methods

Reagents were purchased from Thermo Fisher Scientific unless otherwise stated.

### 2.1 Cell Culture

A549 (ATCC CCL‐185) cells were grown in standard medium (SM) containing Dulbecco's Modified Eagle's Medium (DMEM low glucose) supplemented with 10% (v/v) foetal bovine serum (FBS), 1% (v/v) non‐essential amino acids, 1% (v/v) L‐Glutamine, and 1% (v/v) penicillin/streptomycin. The cells were passaged using 0.25% trypsin/ 0.02% EDTA and maintained at 37°C in 5% CO_2_. Differentiation medium (DM) was prepared using SM medium supplemented with glucose (4.5g/l), 1 µm dexamethasone (Dex, Tocris), 100 µm isobutylmethylxanthine (IBMX, Sigma–Aldrich), 1 µm rosiglitazone (Rosi, Tocris), 10 µg/mL insulin (Ins, Sigma–Aldrich).

### 2.2 Cell Growth Analysis

Cells were seeded in SM or DM at in a 24‐well (day 0) and on days 3, 5, 7, 10 and 15, wells were harvested to perform cell counts using a Burker chamber (3 wells/condition). A similar set of wells was seeded (day 0), fixed at days 3, 5, 7, 10 and 15 to be stained with 0.05% crystal violet and quantified after chemical dye extraction on a plate reader (POLARstar Omega, BMG LABTECH) as described elsewhere [22]. Morphological features such as cell length and vesicle numbers were analyzed using Fiji (NIH).

### 2.3 Cell Cycle Analysis

Propidium iodide (PI) was used to stain DNA as described elsewhere [23], and cell cycle profile was analyzed on a BD FACS Lyrics flow cytometer recording 50 000 events per sample.

### 2.4 Senescence Assay

Cells treated for 4 days in SM or DM were stained with the CellEvent Senescence Green detection kit according to the manufacturer's instructions. Primary fibroblasts treated with 10mm H_2_O_2_ were used as positive control [24].

### 2.5 Lysotracker Staining

Cells treated for 5 days in SM or DM were harvested, counted and seeded on glass coverslips in their respective medium, at a density of 5 × 10^4^ cells/well. In parallel, a sample of SM‐treated cells was treated overnight with 50 µm chloroquine (Sigma–Aldrich) from the following day onward, to provide a positive control [25, 26]. At day 7 of differentiation, coverslips were washed twice with PBS and incubated for 1 h with Lysotracker Green (100 nm in culture medium) and Hoechst 33258 dye (at the final concentration 0.5 µg/mL) to counterstain nuclei. At the end of the incubation, samples were washed twice with PBS before live observation on a LEICA TCS SP8 confocal microscope (CGS, University of Pavia).

### 2.6 Transmission Electron Microscopy (TEM)

Cells were harvested by mild trypsinization with 0.25% trypsin/0.02% EDTA, centrifuged at 150 g for 10 min and then fixed with 2.5% glutaraldehyde and 2% paraformaldehyde in culture medium for 2 h at RT. Following PBS washes, cells were post‐fixed with 1% aqueous osmium tetroxide (OsO4) for 2 h at RT, pre‐embedded in 2% agarose, dehydrated using graded acetone, and finally embedded in epoxy resin (EM‐bed812, Electron Microscopy Sciences, Hatfield, PA, USA). Ultrathin sections (60–80 nm) were cut on a Reichert OM‐U3 ultramicrotome and collected on nickel grids (200 Mesh). The grids were counterstained with uranyl acetate and lead citrate as described [27] and observed with a JEM 1200 EX II electron microscope (JEOL, Peabody, MA, USA) operating at 100 kV and equipped with a MegaView G2 CCD camera (Olympus OSIS, Tokyo, Japan).

### 2.7 Cell Adhesion Assay

Cell adhesion profiles were analyzed with iCELLigence (ACEA Biosciences) following manufacturer's instructions. Briefly, A549 cells pre‐treated for 7 days in SM or DM were then seeded at 10^4^ cells/well in quadruplicates, and after 30 min measurements of cell index (CI) were recorded over 24 h.

### 2.8 Migration Assay

Cells were seeded into 2‐well culture inserts (Ibidi) according to the manufacturer's instructions. The experiment was started (T0) when cells were confluent and the insert removed. Gap closure was imaged every 5 min over 40 h on a Leica DMi8S microscope. Time‐lapse videos (*n* = 6 per condition) were analyzed using Fiji (NIH) with the Wound Healing Size plug‐in (https://github.com/AlejandraArnedo/Wound‐healing‐size‐tool/wiki).

### 2.9 Clonogenic Assay

Cells pretreated for 7 days in either SM or DM were dissociated with 0.25% trypsin/ 0.02% EDTA, re‐seeded in 6‐well plates at 300 cells/well (*n* = 4) and maintained in either SM or DM. After 7 days of treatment, wells were formaldehyde‐fixed and stained with 0.25% crystal violet to analyze colony number and morphology [28] in each condition on a Leica DMIL microscope.

### 2.10 Alkaline Phosphatase (ALP) Assay

ALP activity in samples treated for 7 days with SM or DM was quantified using a Sigmafast kit (Sigma–Aldrich) according to the manufacturer's instructions, and absorbance at 405 nm was measured on a microplate reader (POLARstar Omega, BMG LABTECH) (*n* = 4). ALP staining was performed with the SK‐5400 detection kit (Vector Laboratories) according to the manufacturer's instructions, and imaged on a Leica DMIL microscope.

### 2.11 Fluorescence Immunostaining

Cells cultured on fixed coverslips were processed for immunostaining as previously described [29] using antibodies indicated in Table S1. Stained samples were counterstained with DAPI or Hoechst as indicated, mounted with Vectashield (Vector Laboratories) and imaged on a Leica DM6F wide‐field or Leica SP8 confocal microscope as stated. The corresponding quantification of mean fluorescence intensity (MFI) was performed analyzing 5 images per replicate (*n* = 3) with Fiji‐ImageJ (https://imagej.net/software/fiji/), calculating the ratio between the measured fluorescent signal and the total number of nuclei per field of view. For flow cytometry analysis, cells (2 × 10^6^ cells per sample, biological duplicates) were analyzed live (for CD44 detection) or fixed (for SOX2 detection) on a cytometer (BD FACS Lyric, Becton Dickinson). Gating and data analysis were performed with Floreada (https://floreada.io/) using unstained controls.

### 2.12 Hyper7‐NES Transfection and Intracellular H_2_O_2_ Detection by HyPer7‐NES Imaging

The plasmid for the mammalian expression of cytoplasm‐targeted ultrasensitive hydrogen peroxide indicator HyPer7 for optical imaging (pCS2+HyPer7‐NES) [30] was a generous gift from Vsevolod Belousov (IBCh, Moscow, Russia) (Addgene plasmid #136467; http://n2t.net/addgene:136467 (accessed on June 9, 2022; RRID: Addgene_136467)). Cells at 60%–70% confluency were transfected with HyPer7‐NES (3 µg DNA / dish) as previously described [31]. Images were acquired using a CCD camera (DMK 33UP1300) and collected at 10 fps with IC capture software. H_2_O_2_ was added to the cells at a final concentration of 50 µM. Image processing was performed using Image J.

### 2.13 Western Blot

Total protein was extracted from cells treated for 7 days in SM or DM and western blotting was performed as described elsewhere [32] using antibodies indicated in Table S1. Images were taken on a C600 (Azure Biosystems) and densitometric analysis of the protein bands was performed using Fiji (NIH), using β‐actin signal for normalization.

### 2.14 ALDH Functional Assay

The assay was performed using the AldeGreen ALDH Detection kit (Merck Millipore) according to the manufacturer's instructions, using cells grown for 7 days either in SM or DM. Both control and test samples tubes incubated for 45 min at 37°C protected from light before analysis on a BD FACS Lyric flow cytometer (CGS, University of Pavia). Baseline fluorescence established by inhibiting ALDH activity with DEAB was used to gate ALDH^+^ cells.

### 2.15 Quantitative Real Time PCR

Total RNA was extracted using Trizol and the Total SV RNA purification kit (Promega) according to the manufacturer's instructions, with an additional treatment with the RQ1 RNAse‐free DNAse (Promega) to avoid genomic DNA contamination. Samples were retrotranscribed using the Verso cDNA synthesis kit, and quantitative PCR was performed using the PowerUp SYBR Green Master Mix (Applied Biosystems) on the CFX Connect Real‐Time System (BioRad). Gene expression was determined according to the ΔΔCt method using the primers presented in Table S2, with data calculated from biological and technical triplicates.

### 2.16 Soft Agar Colony Formation Assay

The assay was performed as described elsewhere [33]. Briefly, 6.8 × 10^3^ cells pre‐treated for 7 days in SM or DM were seeded in 0.33% agar onto a pre‐cast layer of 0.6% agar, and maintained covered in their respective medium for 14 days. At the end of the culture period, samples were stained with 0.005% gentian violet for 45 min and imaged taking 20 pictures/condition to count the number of colonies and to measure their diameter. Soft agar samples were prepared in triplicates for each condition, and the experiment was repeated twice.

### 2.17 3D Spheroid Formation

Cells were seeded in SM or DM in 6‐well plates at 1.2 × 10^6^ cells/well, in triplicates, and placed on a shaker set (150 rpm) to prevent adhesion, with medium changed twice weekly. Over 14 days of culture, the samples were imaged at different timepoints, and the shortest spheroid diameter was measured (*n* = 3, at least 25 objects/condition). Samples at day 7 were sedimented using a Cytospin centrifuge (Cyto‐System, Hettich) to prepare slides, which were processed for ALP staining as previously described.

### 2.18 Statistical Analysis

**FIGURE 1:**
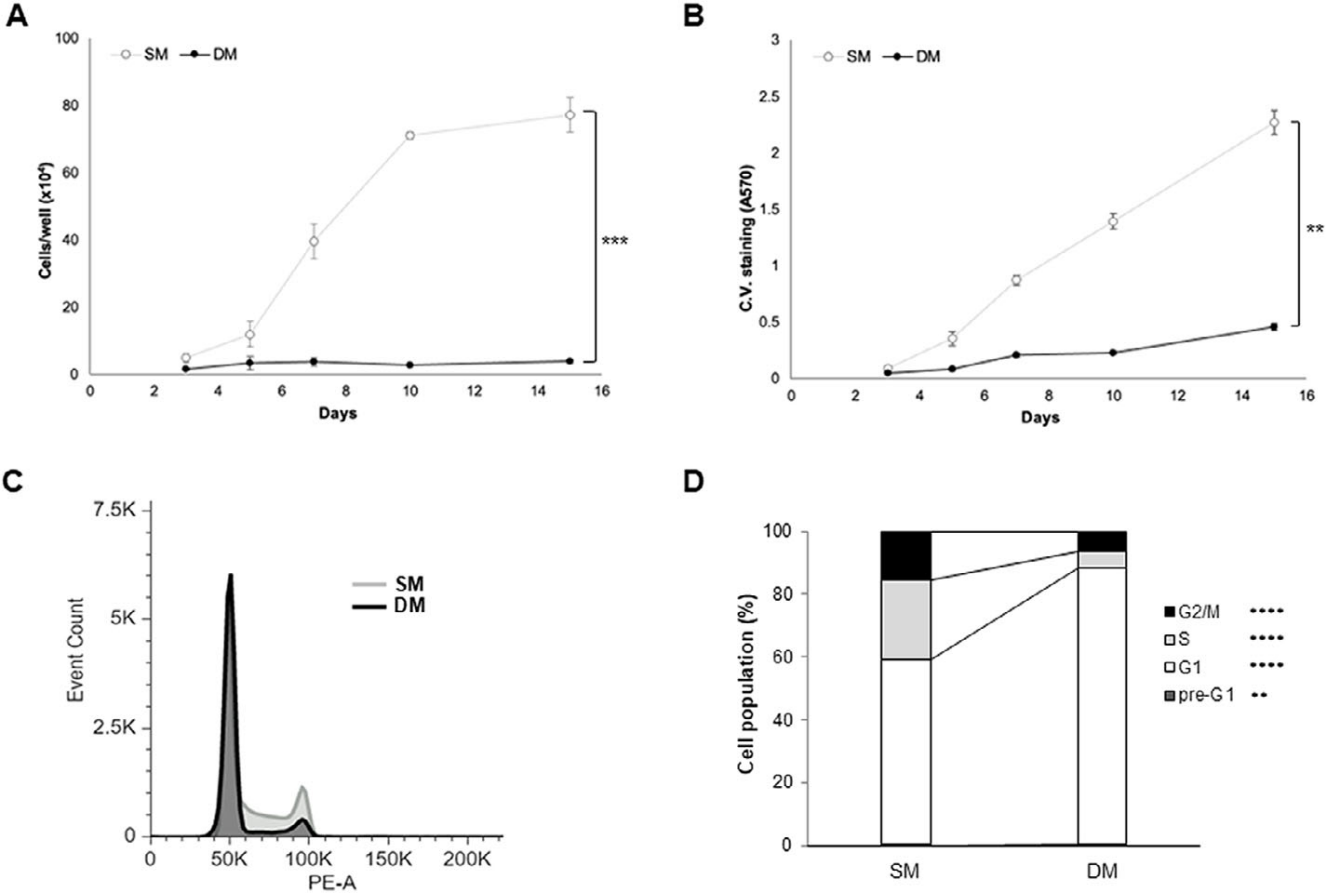
Changes in A549 growth upon exposure to Differentiation medium (DM) over 15 days. (A,B) Cell counts (A) and Crystal violet assay (B) performed over 15 days (*n* = 3, **p < 0.001 measured with a mixed repeated measures Anova test). (C,D) Cell cycle analysis of cultures treated with SM or DM for 7 days showing representative profile (C) and corresponding percentages of the population in the various cell cycle phases (D) after propidium iodide staining (*n* = 3, **p < 0.001, ****p < 0.00001).

**FIGURE 2:**
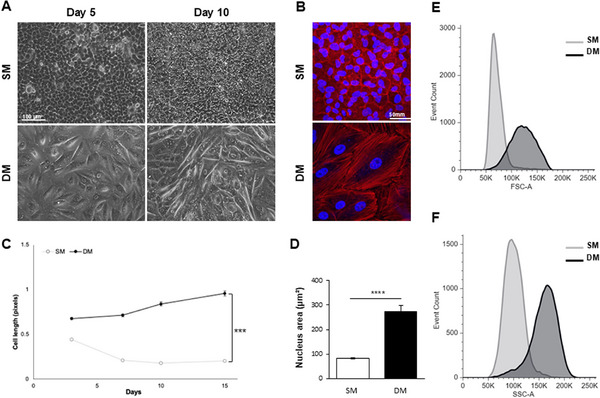
Changes in A549 cell dimensions upon exposure to SM or DM over 15 days. (A) Changes in cell morphology observed in live cultures after 5 and 10 days of treatment. Scale bar: 100 µm. (B) Confocal imaging of phalloidin‐stained (red) cells at day 7 with DAPI nuclear counterstain (blue). Scale bar: 50 µm. (C) Changes in cell length measured over 15 days of treatment staining (*n* = 3, ***p < 0.0001 measured with a mixed repeated measures Anova test). (D) Nuclear area measured in cells treated for 7 days. (E,F) Flow cytometry analysis of cell size (E) and granularity (F) parameters (****p < 0.00001).

Results are represented as mean ± SEM (standard error of the mean) unless otherwise stated. Data was analysed using an unpaired two‐tailed Student's T‐test with the statistical difference between conditions shown for p values as *p < 0.01, **p < 0.001, ***p < 0.0001, ****p < 0.00001. A mixed repeated measures Anova test (Jamovi online free tool, https://www.jamovi.org) was used for time‐course experiments as stated in Figures 1, 2 and Figure S2B, with the Greenhouse‐Geisser and Huynh‐Feldt ℇ corrections, showing statistical difference between conditions for p values as **p < 0.001, ***p < 0.0001.

## 3 Results

### 3.1 Differentiation Medium Reduces Proliferation in A549

In order to assess the response of A549 cells to Differentiation medium, cell growth was assessed over 15 days in cultures maintained in either SM or DM. A significant decrease in cell growth was seen upon exposure to DM (Figure 1A,B) evidenced by cell counts and crystal violet assay. Cell cycle profile analyzed at day 5 confirmed a decrease in proliferative cells under DM conditions, with a significant decrease in S and G2/M phase and corresponding increase in the G0/G1 fraction (Figure 1C,D).

To assess whether the change induced by DM exposure is linked to increased senescence, an assay was carried out and indicated the absence of significant senescence‐associated β‐galactosidase activity in DM‐treated cells (Figure S1).

### 3.2 Differentiation Medium Causes Marked Phenotypical Changes in A549 and the Appearance of Vesicle‐Containing Cells

**FIGURE 3:**
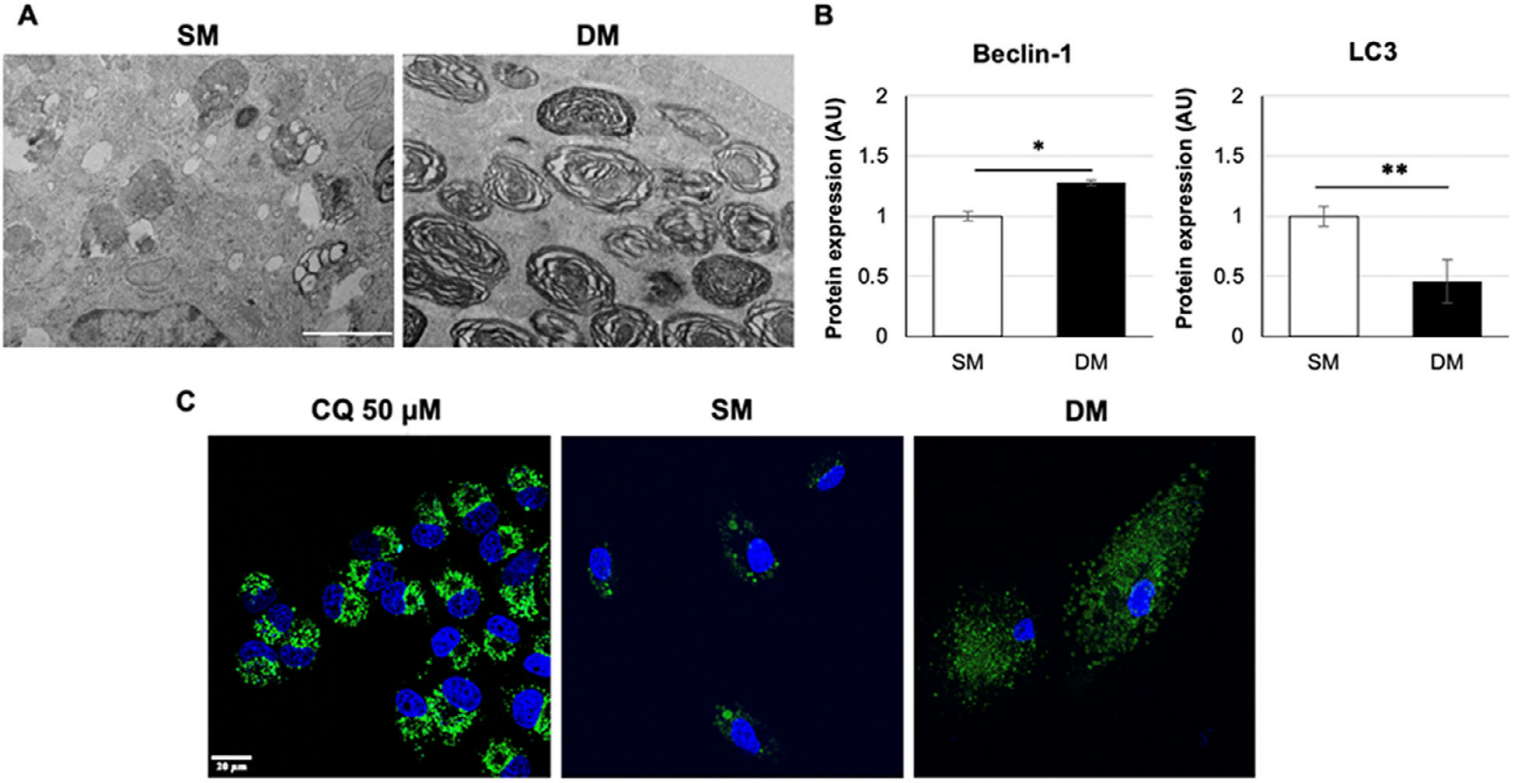
Induction of vesicle‐containing cells upon exposure to Differentiation medium. (A) TEM images of A549 cells treated for 7 days with SM or DM. Scale bar: 1 µm. (B) Western blot analysis of Beclin‐1 and LC3 expression after 7 days of treatment in SM or DM (*p < 0.01, **p < 0.001). (C) Lysotracker Green‐DND26 live staining (green) of A549 cells treated for 7 days in SM or DM, visualized alongside a sample treated with 50 µm CQ used as positive control, with Hoechst 33258 nuclear counterstain (blue). Scale bar: 20 µm.

The cultures maintained in DM exhibited a clear change in appearance visible already at day 3, with a notable increase in cell footprint and length (Figure 2A,C). Significant cell size increase in DM conditions was confirmed by analyzing FSC (forward scatter channel) values by flow cytometry, with values of the SSC (side scatter channel) also indicating a clear increase in cell granularity in DM compared to SM (Figure 2E,F), in line with observed appearance of intracytoplasmic lamellar vesicles in DM‐treated cells (Figure 3A).

To investigate whether these phenotypic changes were solely due to the presence of Dex in DM, cells were treated with DM or Dex only, which produced a significantly lower response compared to DM (Figure S2A,B). To determine whether the vesicles observed may be lipid droplets typical of adipocytes, staining with the lipophilic dye Oil Red O was performed (Figure S2C). The visible lack of staining indicated that the vesicles were not lipid‐filled. The structure of these vesicles investigated by Transmission Electron Microscopy (TEM) showed that DM samples were observed to contain numerous multilamellar bodies (MLBs) and multivesicular bodies (MVBs), a feature possibly associated both with Alveolar type II cell differentiation and with cellular stress (Figure 3A). Analysis of autophagic protein markers Beclin‐1 and LC3 expression (Figure 3B; Figure S3) showed increased Beclin‐1 following DM exposure but significantly lower LC3 compared to SM conditions, suggesting incomplete autophagic induction. Lysotracker staining was used to investigate whether appearance of lamellar vesicles in DM‐treated cells was associated with increased lysosomal trafficking. The images collected (Figure 3C) showed a visible signal increase in DM compared to SM, corresponding to an accumulation of lysosomes‐derived vesicles, although larger vesicles corresponding to those observed in TEM and brightfield microscopy were devoid of Lysotracker signal.

### 3.3 Differentiation Medium Increases Differentiation Markers and Reduces Cancer Features

**FIGURE 4:**
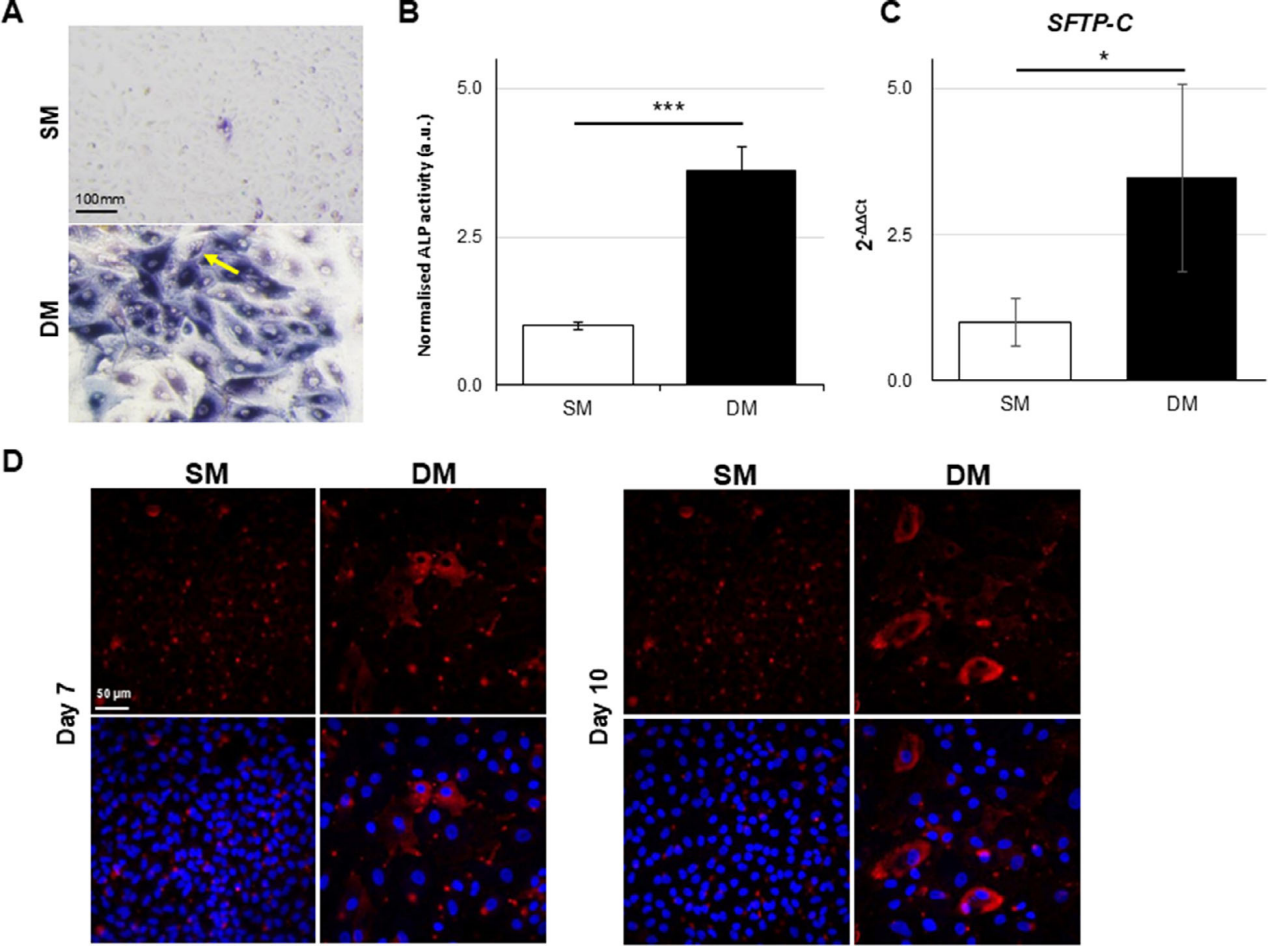
Differentiation features upon DM treatment. (A) Alkaline phosphatase (ALP) activity visualized at day 7 by histological staining. Scale bar: 100 µm. (B) ALP enzymatic assay measured at day 7 and normalized to Crystal violet signal for cell content. (C). Gene expression of SFTP‐C after 7 days in SM or DM analyzed by RT‐qPCR (*p < 0.01, ***p < 0.0001). (D) Immunostaining showing SFTP‐C signal (red) with DAPI (blue) used as nuclear counterstain in A549 treated for 7 and 10 days. Scale bar: 50 µm.

To test whether the vesicles formed under DM may be indicative of an alveolar phenotype, the level of alkaline phosphatase (ALP) activity, a marker associated with alveolar differentiation [34], was measured in cell samples. Cells treated in DM showed a significant increase in ALP enzymatic activity at 7 and 10 days compared to SM‐treated cells (Figure 4A,B). RT‐qPCR analysis of the alveolar marker Surfactant Protein C (SFTP‐C) confirmed a significant increase in gene expression levels (Figure 4C) after 7 days in DM, in line with SFTP‐C immunostaining, which showed vesicular signal at both 7 and 10 days (Figure 4D).

**FIGURE 5:**
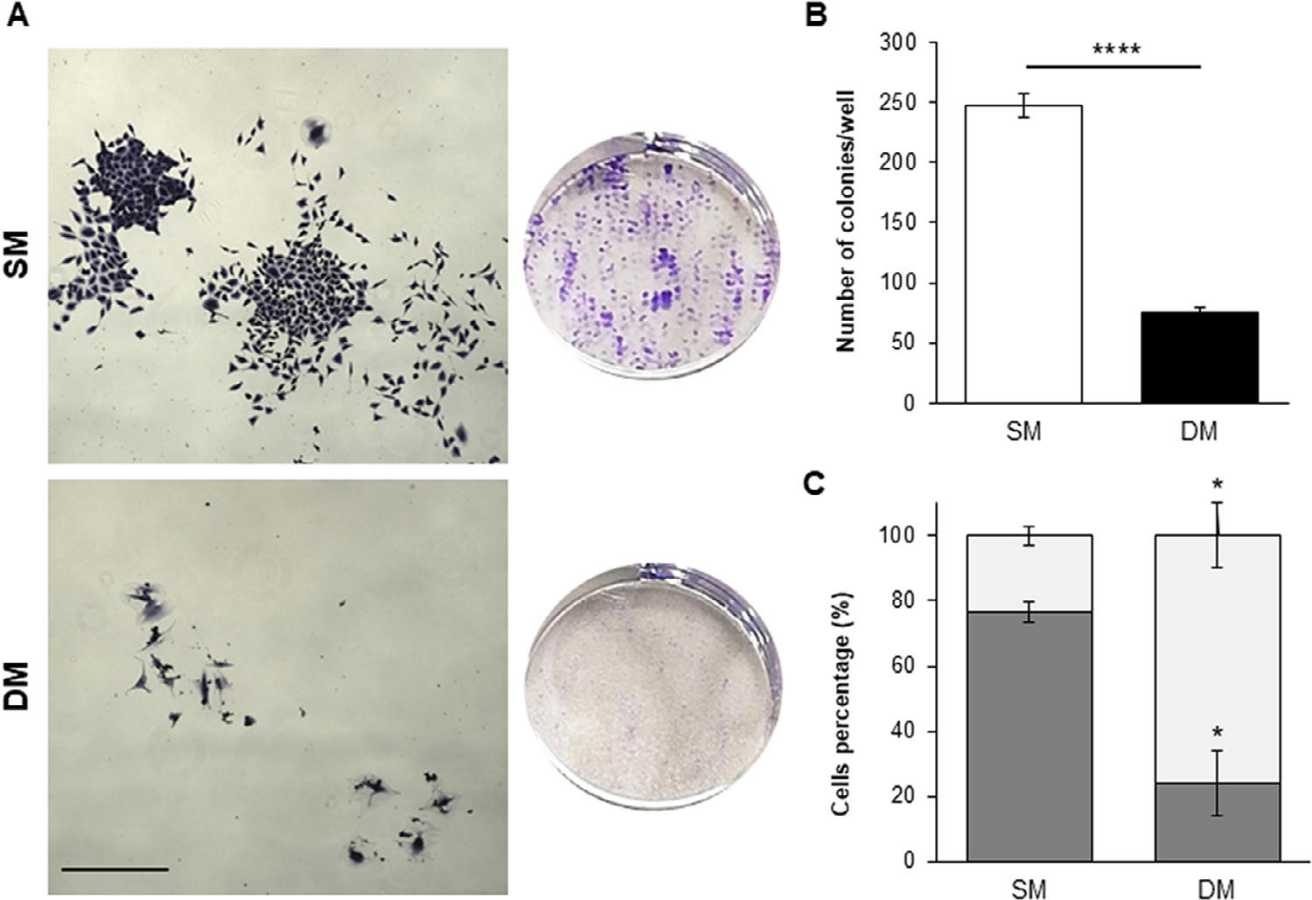
Changes in clonogenicity upon DM treatment. (A) Representative images of stained colonies (left) formed in 6‐well plates (right) Scale bar: 200 µm. (B) Quantification of the number of colonies formed. (C) Relative percentages of undifferentiated (dark gray) vs differentiated (light gray) colonies under the two culture conditions. (*p < 0.01, ****p < 0.00001).

**FIGURE 6:**
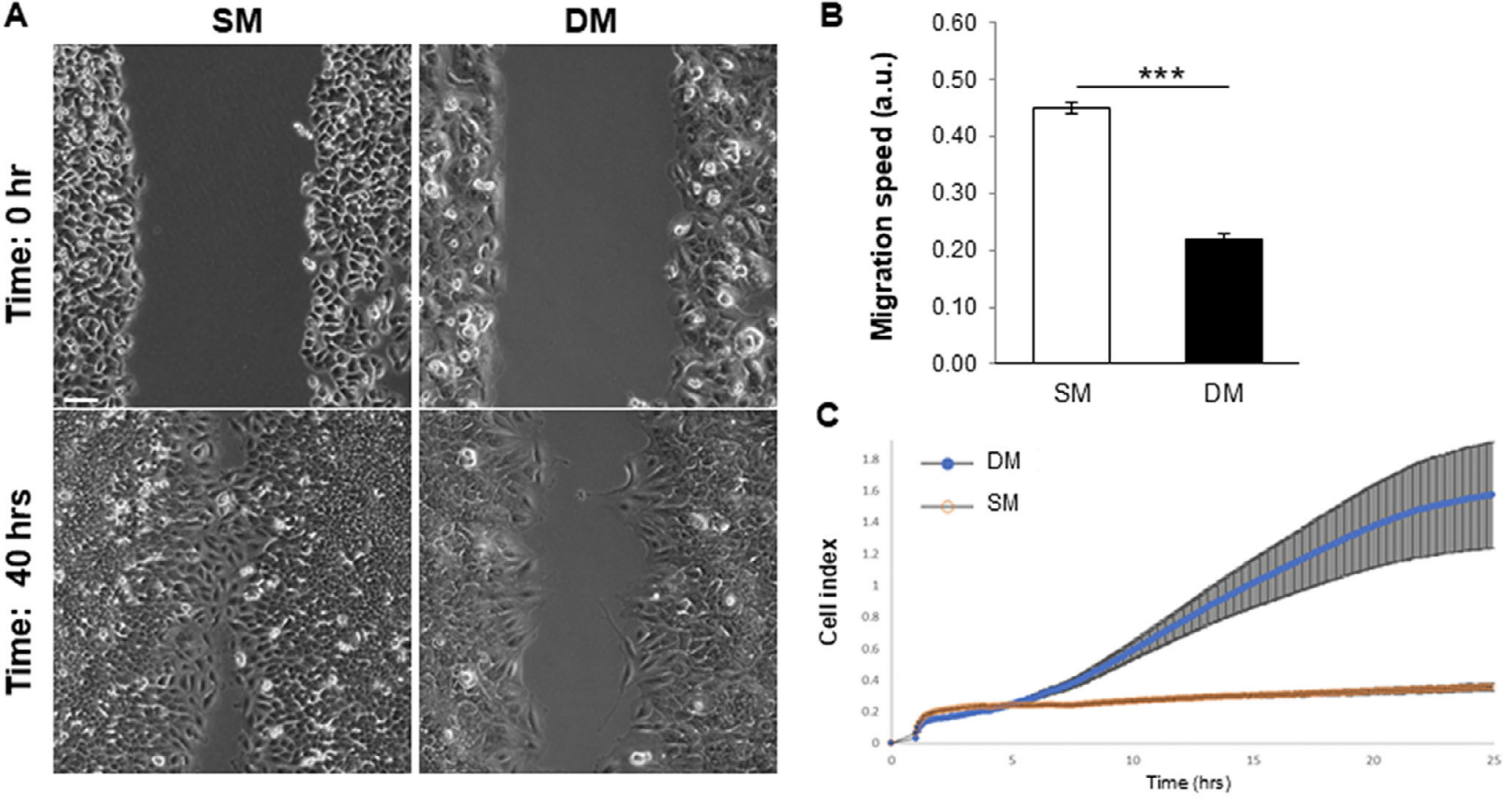
Decreased A549 migration and increased A549 adhesion upon DM treatment for 7 days. (A) Representative images of the gap at the start of the assay and after 40 h. Scale bar: 100 µm. (B) Front migration speed measured over 40 h (*n* = 6, ****p < 0.00001). (C) A549 adhesion properties measured through iCELLigence technology for 24 h, after 7 days of exposure to SM or DM (****p < 0.00001).

A clonogenic assay was performed with cells treated for 14 days in either SM or DM, in order to further characterize the phenotypic change and evaluate its impact on clonogenicity, a property reflective of cancer stem cells [35]. The number of colonies formed was found to drastically decrease for DM‐treated cells (Figure 5A,B), which demonstrated a shift from holoclone to paraclone appearance when compared to SM‐treated controls (Figure 5C). The phenotypic changes were also assessed in terms of migration ability in a gap closure assay, which indicated a significant reduction in cell migration after 7 days in DM conditions (Figure 6A,B). This alteration was consistent with the outcome of an impedance‐based analysis, which indicated an increase in cell adherence in DM‐treated cells compared to SM‐treated controls (Figure 6C).

**FIGURE 7:**
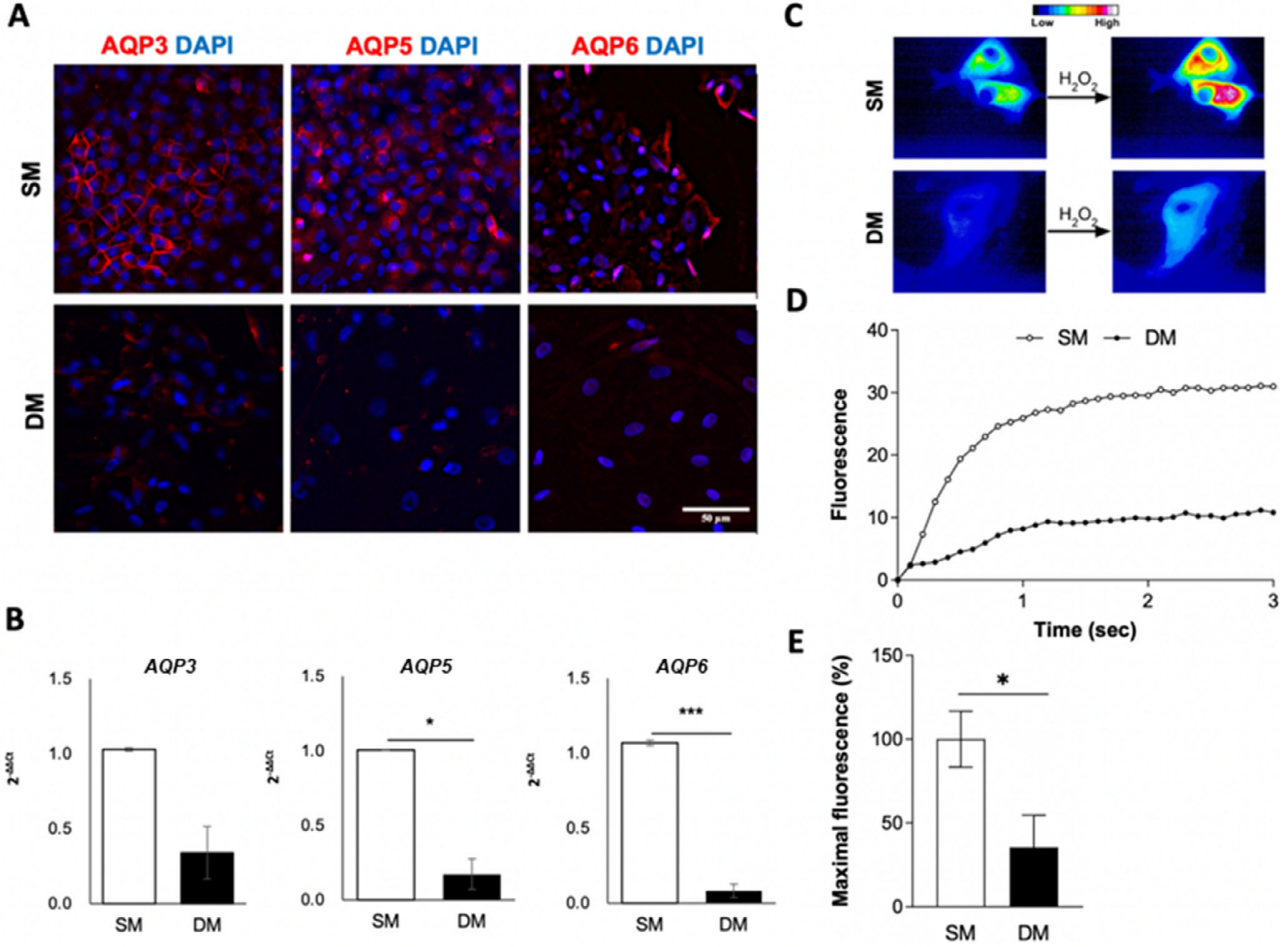
AQP expression and activity in A549 cells treated in SM or DM. (A,B) Expression of AQP3, AQP5 and AQP6 in cultures after 7 days in SM or DM analyzed by immunodetection (A, red) and RT‐qPCR (B). Hoechst 33258 dye (blue) used for nuclear counterstain in (A). Scale bar: 50 µm. (C) Functional analysis of AQP activity after 7 days in SM or DM using the HyPer7 fluorescent reporter, to measure the kinetics of H_2_O_2_ permeability (D) and corresponding quantification (E). (*p < 0.01, ***p < 0.0001).

Changes in aquaporin (AQP) expression, associated with CSCs and cancer cell migration [36], was assessed by immunodetection and RT‐qPCR (Figure 7A,B), indicating a decrease in AQP3, AQP5 and AQP6 gene and protein expression upon DM treatment. This was further confirmed using a fluorescent reporter for AQP function measuring the kinetics of H_2_O_2_ permeability, which demonstrated a significant reduction in AQP activity for DM‐treated cells compared to SM controls (Figure 7C,D).

### 3.4 Differentiation Medium Decreases CSC Features in A549

**FIGURE 8:**
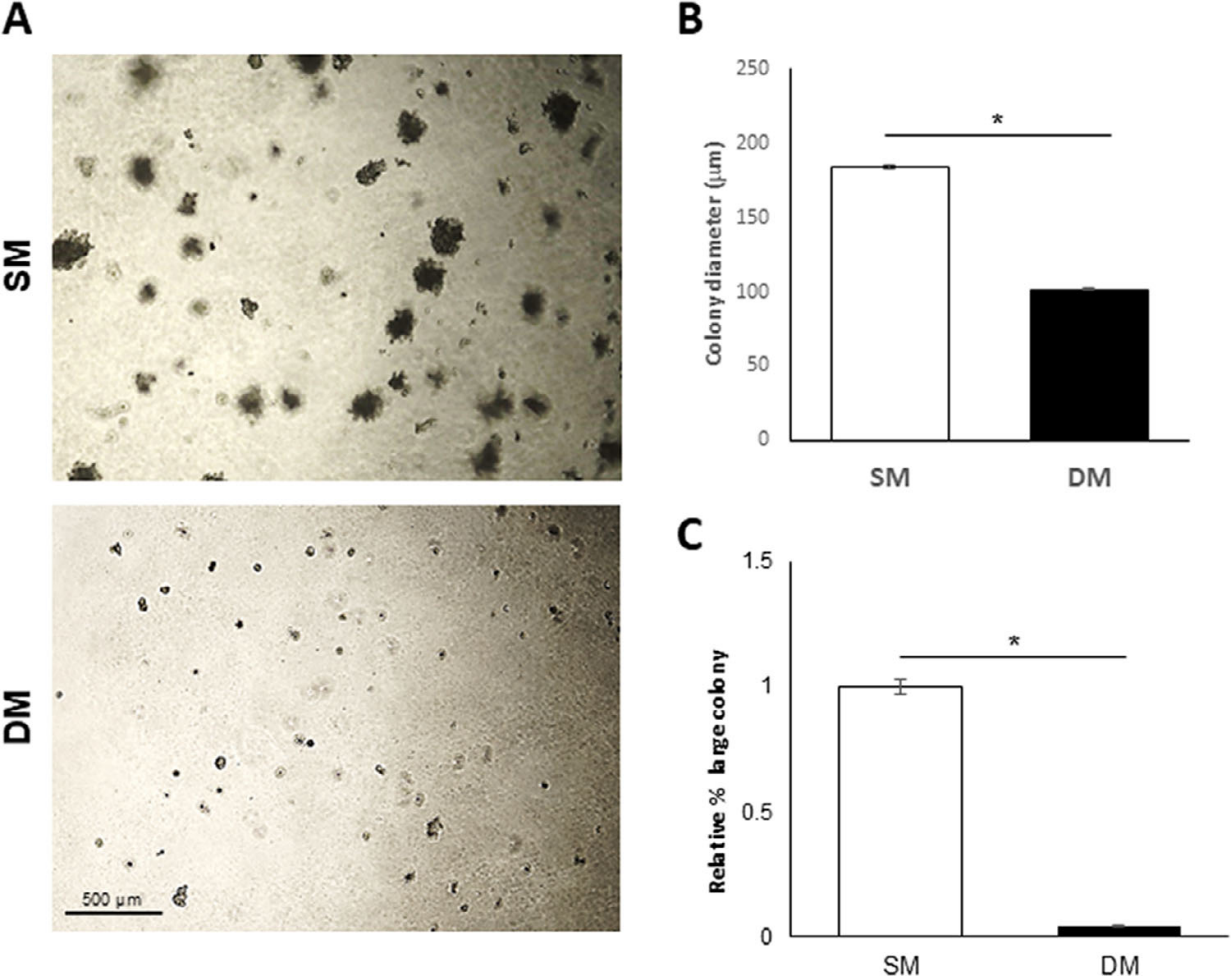
Soft agar colony formation assay for A549 cells in SM or DM conditions. (A) Images of colonies obtained after 14 days in soft agar. (B,C) Corresponding analysis of (B) colony formation efficiency and (C) colony size compared to SM control measured after 14 days in 2 independent experiments (*n* = 3 each, *p < 0.01). Scale bar: 500 µm.

To further probe the hypothesized decrease in CSC potential in response to DM treatment, anchorage‐independent colony formation was measured with a soft agar colony formation assay performed with cells pre‐treated with SM or DM for 7 days and monitored over 14 days in their respective media. Colony quantification (Figure 8) showed a significant inhibition of anchorage‐free colony formation under DM conditions compared to SM controls.

**FIGURE 9:**
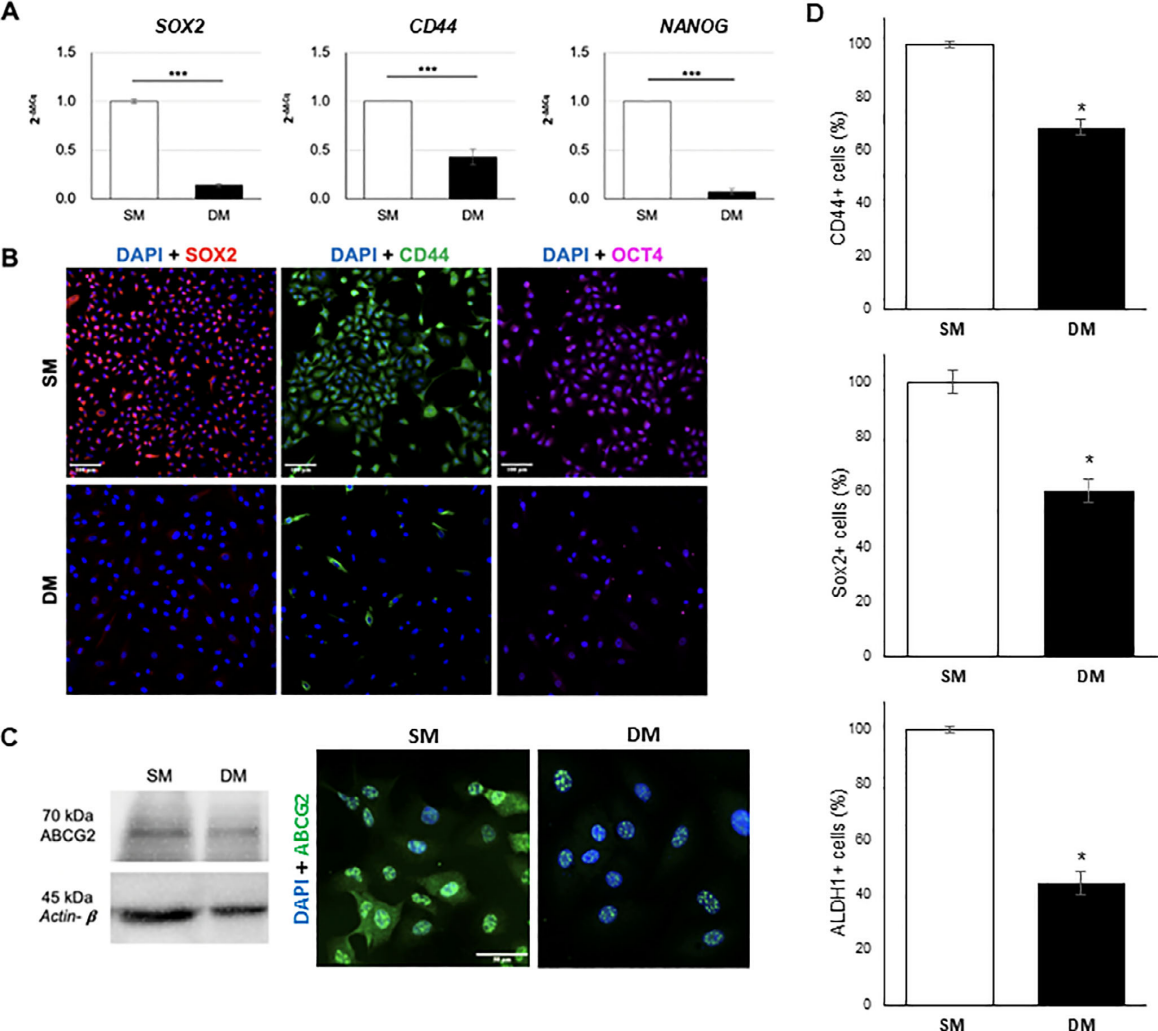
Changes in CSC markers expression in A549 cells upon 7‐day DM treatment. (A) RT‐qPCR analysis of SOX2, CD44 and NANOG (***p < 0.0001). (B) Immunostaining for SOX2 (red), CD44 (green), and OCT4 (purple) with DAPI counterstain (blue). Scale bar: 100 µm. (C) ABCG2 expression analyzed by Western Blotting and immunofluorescence (green), with DAPI counterstain (blue). Scale bar: 50 µm. (D) CD44+, SOX2+ and ALDH1+ cell populations measured by flow cytometry in DM compared to SM (*p < 0.01).

The expression of prominent CSC markers was analyzed in SM and DM‐treated cultures at day 7. A significant decrease in SOX2, CD44 and NANOG gene expression (Figure 9A) was observed, as well as a reduction in SOX2, CD44 and OCT4 protein levels in DM‐treated samples compared to SM controls (Figure 9B). Although the level of ABCG2 protein expression was only mildly affected by DM treatment (Figure 9C), its presence on the plasma membrane was lost in treated cells, which presented an entirely nuclear expression, unlike the SM control in which expression was both membranous and nuclear. DM treatment was also seen to reduce the percentage of cells positive for ALDH activity, another CSC feature, when compared to SM (Figure 9D; Figure S4 and S5).

**FIGURE 10:**
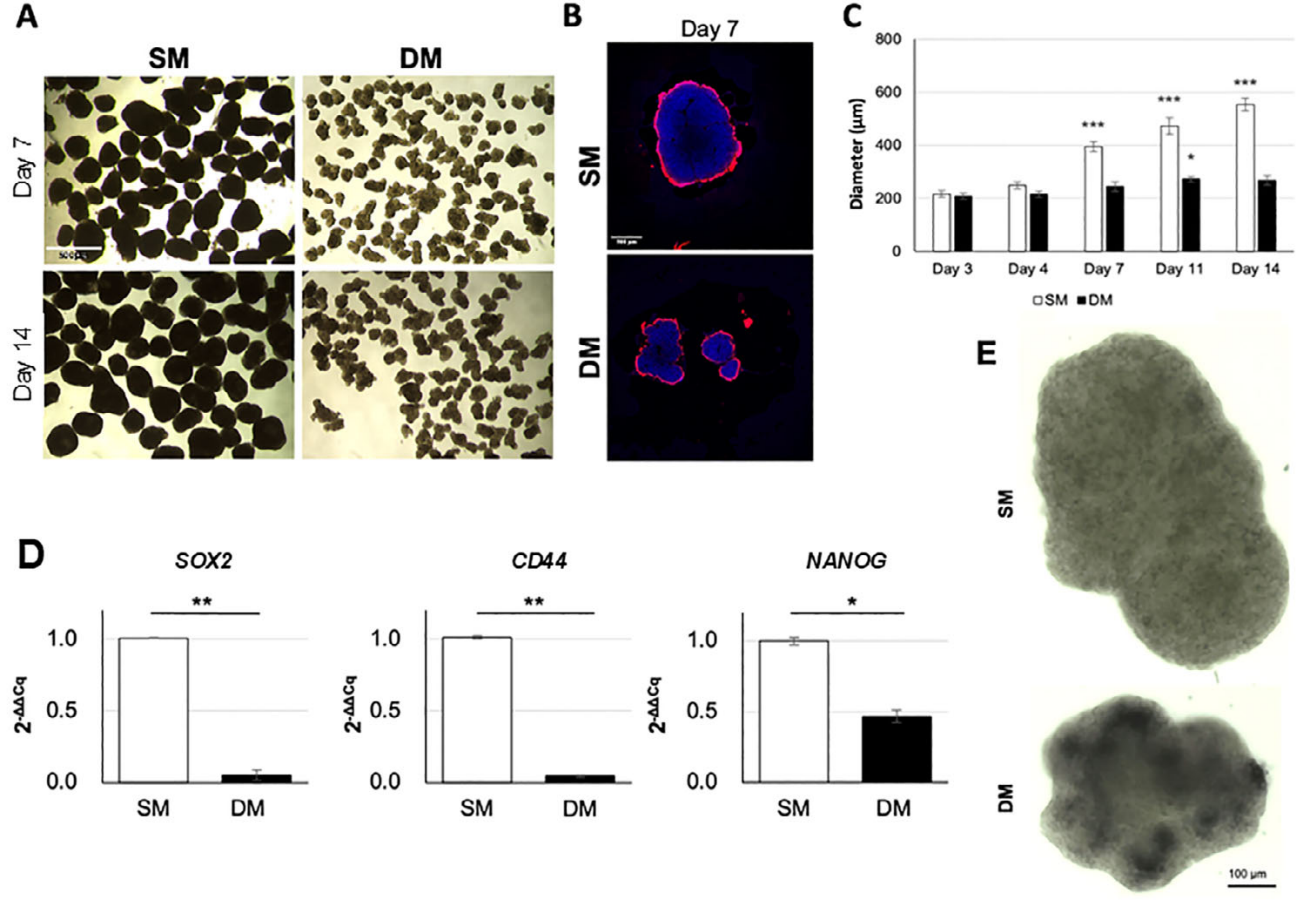
Spheroids formation in 3D cultures of A549 in SM or DM. (A) A549 spheroids at day 7 (top) and 14 (bottom). Scale bar: 500 µm. (B) Phalloidin (red) staining of day 7 spheroids in SM or DM medium, with DAPI nuclear counterstain. Scale bar: 500 µm. (C) Spheroid diameter measured over 14 days. (D) RT‐qPCR expression analysis of stem cell markers SOX2, CD44, and NANOG at day 7. (E) ALP staining (dark blue) in spheroids processed by Cytospin. Scale bar: 100 µm. (*p < 0.01, **p < 0.001, ***p < 0.0001).

Finally, the ability to grow as 3D spheroids, a feature of CSCs reflecting stemness [37], was evaluated in suspension cultures. Over time, a notable reduction in spheroid growth and sphericity was observed in DM conditions compared to SM, which grew in size over 14 days with cavities visible from day 10 onward (Figure 10). Expression of stem cell markers SOX2, NANOG and CD44 was found to be down‐regulated in DM‐cultured spheroids (Figure 10D), while conversely, ALP staining performed on Cytospin samples from 7‐day spheroids, as previously done with monolayer cultures, confirmed the positive signal for this differentiation marker in DM‐treated samples (Figure 10E).

## 4 Discussion

### 4.1 Differentiation Approach

In the present study, treatment of A549 adenocarcinoma cells with a differentiation medium containing IBMX, Dexamethasone, Insulin and rosiglitazone resulted in a severe phenotypical change and the near‐ablation of features such as proliferation, migration, clonogenicity and 3D spheroid formation, indicative of aggressiveness. In addition, expression of CSC markers was notably reduced, while differentiation‐linked ALP activity and SFTP‐C expression increased. A similar approach used successfully to differentiate breast cancer cells [21] provided the grounds for the present hypothesis that pro‐adipogenic treatment may be able to alter the cancer phenotype of a NSCLC model tested here with A549 cells.

### 4.2 DM Triggers Differentiation Response but Not Adipogenesis

Of note, and contrary to the starting hypothesis based on previous reports [38, 39], an adipogenic response was not observed in A549 cells: while treatment with DM containing pro‐adipogenic components such as Dex, IBMX, insulin and rosiglitazone, microscopy analysis evidenced the accumulation of intracellular vesicles, in the present model these vesicles remained Oil Red O‐negative throughout the treatment and were visibly different from adiposomes. The absence of neutral lipid droplets was further supported by TEM analysis, which confirmed the presence of numerous lamellar bodies under DM treatment. Rather than adipogenesis, the present experimental results are consistent with the formation of type II pneumocytes [40, 41], known to contain such lamellar bodies [42], in response to DM. This is in contrast with the aforementioned reports that in vitro the use of medium supplemented with PPARgamma ligands [38] or Dex [39] may trigger adipogenesis in A549 cells, inferred from the accumulation of lipid droplets. Here, increased cytoplasmic lysotracker signal observed in DM samples, which can be linked to an autophagic response but also to alveolar lineage [43], together with the positive SFTP‐C staining [44, 45, 46, 47], a pneumocyte II marker [48, 49, 50], hinted to the advent of alveolar differentiation. This was further supported by the induction of SFTP‐C at the transcript level and the increase in alkaline phosphatase activity upon DM treatment, two well established alveolar type II markers [34, 51].

The observed phenotypic response to DM treatment might be explained by the presence of Dex, as glucocorticoids have been observed to decrease proliferation in A549 without triggering senescence [40], and increase alveolar maturation and surfactant production in vivo [52, 53] and in vitro [54]. In the present study, however, exposure to Dex alone could not account for the phenotype observed under DM, which required the complete medium formulation, pointing to a combined effect of DM components. The DM‐induced decrease in proliferation was not associated with increased senescence, which would be consistent with evidence from the literature showing a promoting effect for some of the DM components on alveolar maturation used individually or in pairs, as reported for insulin, dexamethasone, compounds such as IBMX causing cAMP increase, and PPARgamma ligands [55, 56, 57, 58] While a previous report suggested that A549 may be unresponsive to differentiation in the presence of insulin and dexamethasone alone [59], a combination of Dex and high cAMP level was observed to promote SFTP‐C expression in human fetal lung explants [60], in agreement with the present observations for the Dex‐ and IBMX‐containing DM treatment used here.

### 4.3 Decrease in CSC Features

Considering the promotion of A459 differentiation observed upon DM treatment, possible changes in stemness features were analyzed in terms of clonogenicity, anchorage‐independent growth, and expression levels of molecular markers associated with CSCs such as SOX2, NANOG, CD44 [61]. Indeed, DM‐treated cells displayed poor clonogenic potential compared to untreated controls, as well as reduced anchorage‐independent capacity, a feature typical of CSCs. Expression of SOX2, NANOG, CD44 and OCT4 was substantially reduced upon DM treatment, supporting a decrease in CSC markers [62]. ALDH enzymatic activity, another cellular feature common to stem cells and CSCs [63, 64], was found to be negatively affected by DM treatment, showing significant reduction. Likewise, expression of ABCG2, associated with drug resistant populations and known to be expressed in NSCLC [65], was found altered both in terms of reduced expression level and pattern of expression, losing its membranous component in favor of a nuclear signal. These results are in line with previous reports of nuclear ABCG2 shuttling in lung cancer resulting in reduced cell migration and increased differentiation [65, 66], with consequently a decreased ABC transporter efflux activity at the cell membrane expected to reduce drug resistance [67]. Finally, the capacity to form spheroids in suspension cultures, a parameter indicative of CSCs [37, 68], was also markedly reduced after DM exposure.

Altogether, these results indicate that DM treatment resulted in a marked reduction in cellular and molecular features associated with CSCs, in addition to and fitting with the pro‐differentiation effect discussed above. Furthermore, the substantial reduction by DM on A549 migration was equally informative, possibly indicative of a lower metastatic capacity [69]. This would also be in line with AQP measurements, which confirmed lower overall activity in DM as well as significant downregulation of membranous AQP3, AQP5 and AQP6 expression, all linked to cell migration and tumor progression in the context of cancer and NSCLC [31, 36, 70, 71], with AQP3 considered an unfavorable lung adenocarcinoma marker [72]. The decrease in CSC features under DM was likely reinforced by the presence of the PPARgamma ligand rosiglitazone, considering the role of PPARgamma in reducing tumorigenic features such as soft‐agar colony growth observed in overexpression studies performed in H2122 lung cancer [73], and colon cancer [74] models, so it would be of interest to test alternative analogues or ranges of concentrations. In vivo, the protective effect of PPARgamma overexpression on tumor progression has been tested in a rat NSCLC xenograft model [73], however, in this study, PPARgamma overexpression was seen not to be sufficient to inhibit NSCLC migration, while here exposure to the full DM treatment was seen to significantly reduce cell migration, pointing to a broader effect of the full medium.

## 5 Conclusion and Perspectives

Overall, these results indicate a substantial decrease in pathogenic characteristics after exposure of A549 cells to the pro‐differentiation treatment in vitro, as evidenced by a marked reduction in proliferation rates, clonogenicity and migration. Expression of CSC markers and spheroid formation capacity were also reduced, while markers of differentiation significantly increased as a result of DM exposure. These observations arise from a single in vitro model, and as such they now call for extensive follow‐on testing in additional lung cancer cell models, possibly including primary cultures isolated from tumor samples, to extend their clinical and translational applicability. These early results point out how such pro‐differentiation treatment may represent a valuable option worth of further preclinical testing, for instance through optimization of the doses and supplements included, as well as a longer‐term treatment time‐frame. The next steps could also include further analysis of phenotypic changes of the CSC fraction specifically under DM, to assess whether this particular subpopulation could be more directly targeted for therapeutic benefit. In addition, it will now be important to assess the efficacy of the treatment in more complex environments such as 3D tumor models and patient‐derived tumoroids [75], as a means to foresee the likely response in vivo and anticipate the possible challenges of targeted delivery to tumor cells. One potential avenue could involve functionalized nanocarriers, which have recently been developed for lung cancer [76, 77, 78]. Based on the present results, the possible added effect of DM when combined to frequently used chemotherapeutic molecules warrants further investigation, for instance by testing concurrent or sequential administration, as a way to reduce CSC features in NSCLC and other cancer cell lines.

## Author Contributions

A.G., P.F., A.A., K.S., I.O., M.C., G.P., and V.S. were involved in experimental work and data analysis; M.S., M.B., and U.L. provided relevant methodology and experimental resources; V.S. supervised the study; A.G. and V.S. wrote the manuscript with input from co‐authors.

## Funding

This work was supported by the Italian Ministry of University and Research (MUR) through the PhD program in Translational & Precision Medicine and a grant to the Department of Molecular Medicine of the University of Pavia under the initiative ‘Dipartimenti di Eccellenza (2023–2027)’.

## Conflicts of Interest

The authors declare no conflicts of interest.

## Ethics Approval

This article does not contain any studies with human or animal subjects.

## Supporting information




**Supporting File**: adbi70078‐sup‐0001‐SuppMat.docx

**Supporting Table**: adbi70078‐sup‐0002‐SuppTables.docx

## Data Availability

The data that support the findings of this study are available from the corresponding author upon reasonable request.
