# Supplementary material for: Differentiation Treatment Applied to Lung Cancer Model Reduces Pathogenic Traits in Vitro
Source: Adv Biol (Weinh). 2025 Nov 29;10(1):e00371. doi: 10.1002/adbi.202500371 (PMC12798697; doi:10.1002/adbi.202500371)
Supplement: Supplementary file 1 — Supporting File: adbi70078‐sup‐0001‐SuppMat.docx [file ADBI-10-e00371-s001.docx]

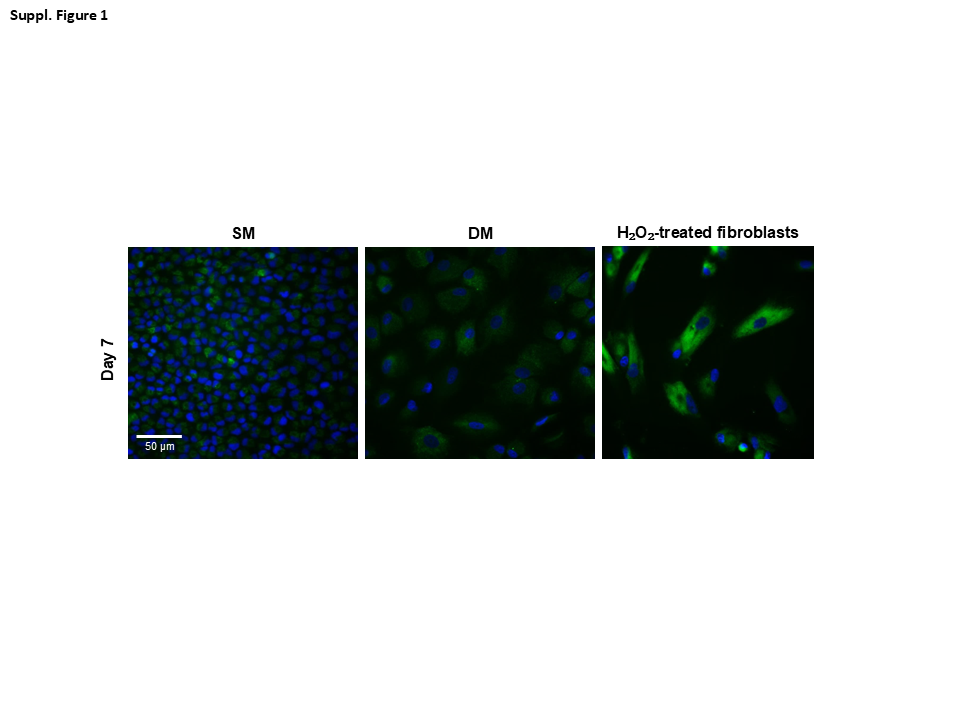


**Suppl. Fig.1: Senescence assay in A549 cells treated for 7 days in SM or DM medium.** Senescence detection assay (green) in cells counterstained with DAPI (blue), alongside human fibroblasts treated with 20 µM H_2_O_2_ used as positive control. Scale bar: 50μm.


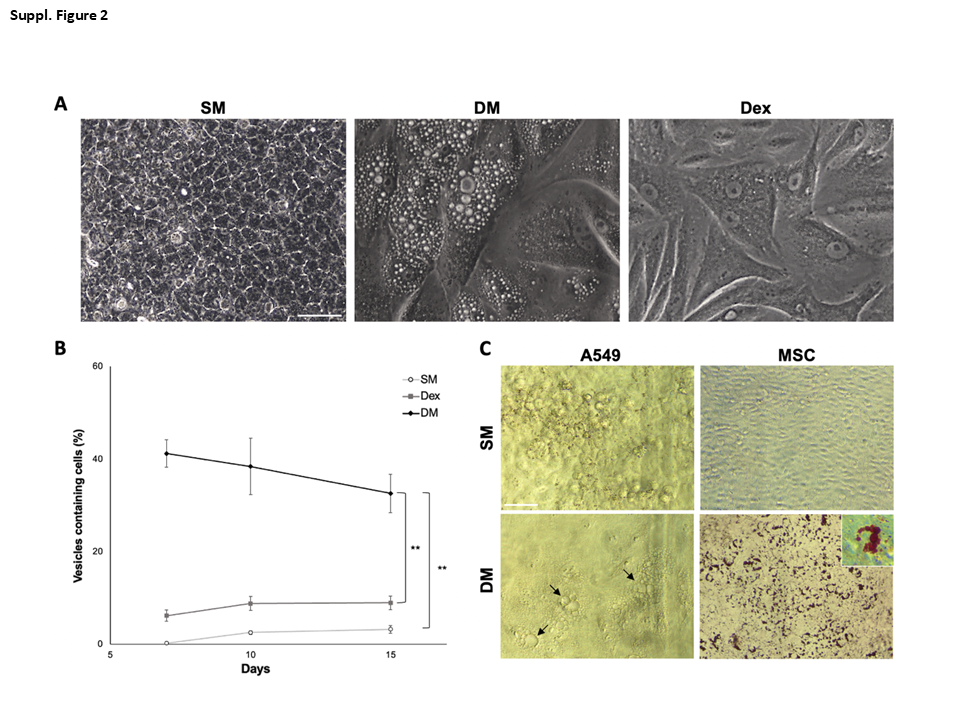


**Suppl. Fig.2: Analysis of vesicle-containing cells upon exposure to DM medium.** (A) Cultures imaged at day 10 of treatment. Scale bar: 50 µm. (B) Percentage of vesicle-containing cells measured at day 7, 10 and 15 of treatment (5 pictures/condition, **p<0.001 measured with a mixed repeated measures Anova test). (C) Oil Red O staining at day 7 showing absence of lipids in the vesicles from DM-treated A549 cells (arrows), compared to the adipogenic cell control (mouse MSCs) showing positive red signal (insert). Scale bar: 100 µm.


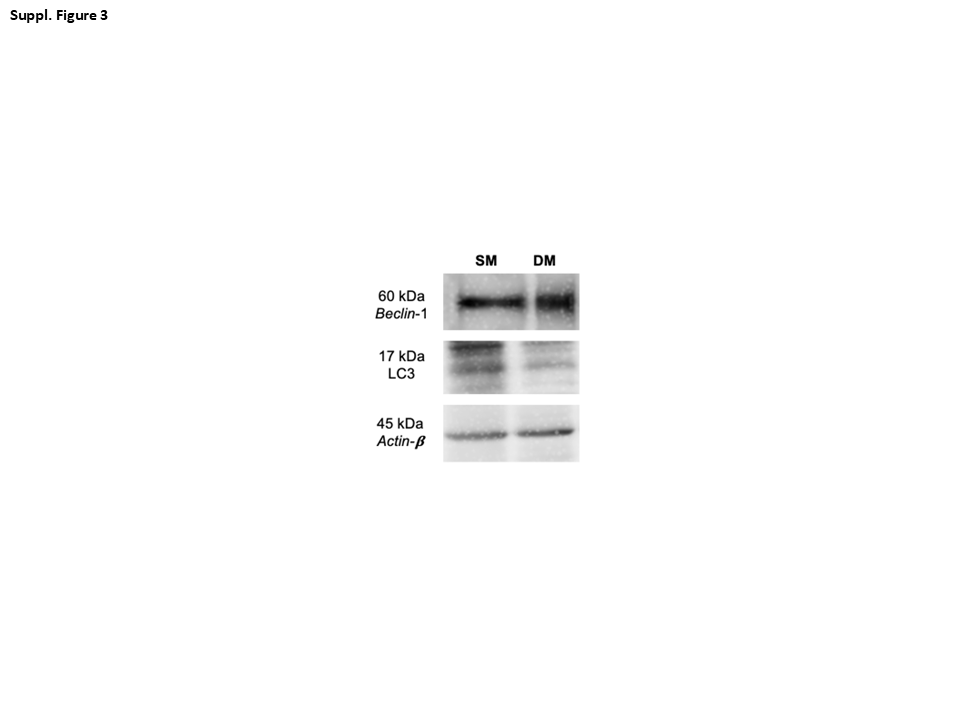


**Suppl. Fig.3: Representative Western blot membrane of Beclin-1 and LC3 expression in cells upon exposure to SM or DM medium.** Actin-β used as loading control.


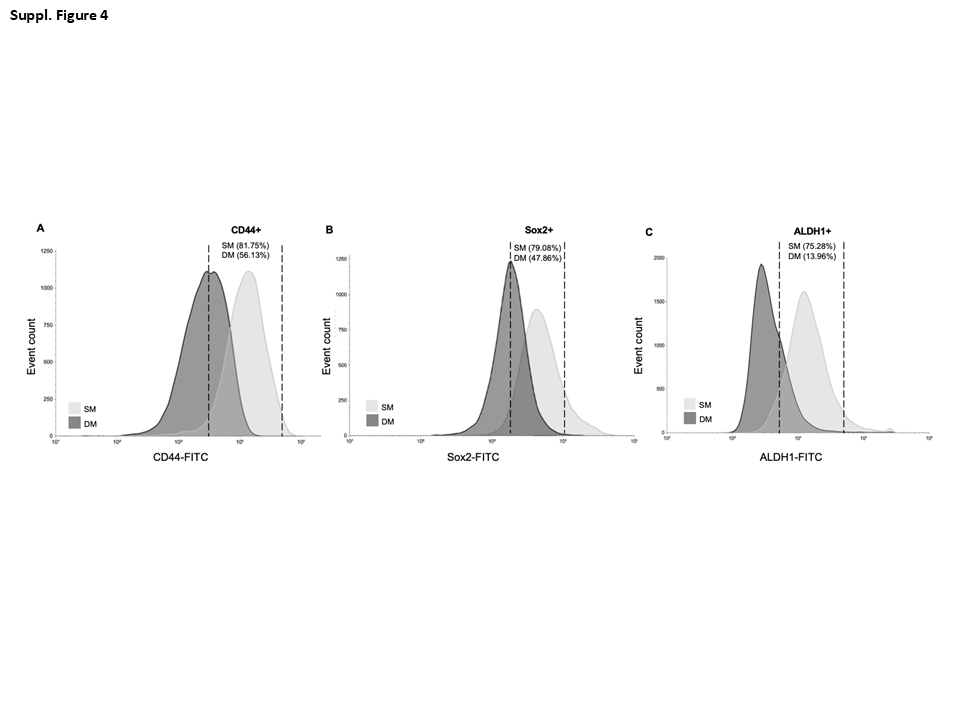


**Suppl. Fig. 4:** Representative flow cytometry histograms of CD44+ (A), Sox2+ (B) and ALDH1+ (C) cell populations in A549 cells treated for 7 days in SM (light grey) or DM (dark grey).


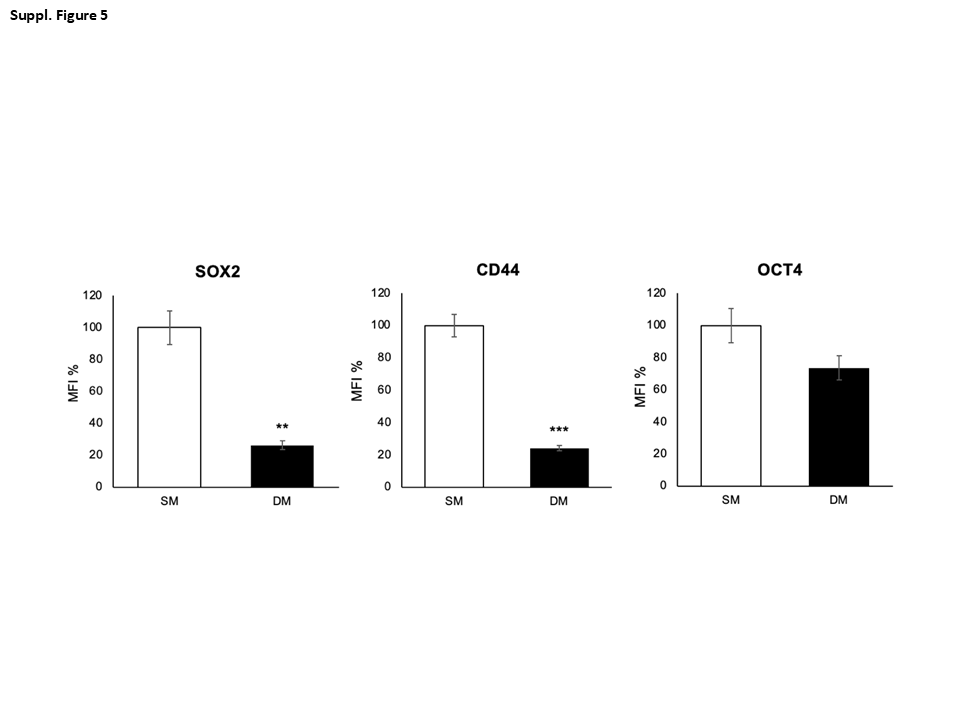


**Suppl. Fig. 5**: Quantification of fluorescence signal for SOX2, CD44 and OCT4 from immunostaining images of 7-day treated DM samples, normalized to SM.
