# Supplementary material for: Differentiation Treatment Applied to Lung Cancer Model Reduces Pathogenic Traits in Vitro
Source: Adv Biol (Weinh). 2025 Nov 29;10(1):e00371. doi: 10.1002/adbi.202500371 (PMC12798697; doi:10.1002/adbi.202500371)
Supplement: Supplementary file 2 — Supporting Table: adbi70078‐sup‐0002‐SuppTables.docx [file ADBI-10-e00371-s002.docx]

**Suppl. Table 1:** antibodies used

| SOX2 | Rabbit, 1:500 If or 1:1000 WB, #ab97959 Abcam |
| --- | --- |
| OCT4 | Rabbit, 1:500 If or 1:1000 WB, #ab19857 Abcam |
| CD44 | Rat, FITC-conjugated, 1:2000 #11-0441-82 Thermo Fisher Scientific |
| SPC | Rabbit, 1:250 IF or 1:1000 WB, #BS-10067R Thermo Fisher Scientific |
| ACTIN | Rabbit, 1:5000 WB, #MA5-15739 Thermo Fisher Scientific |
| AQP3 | Rabbit, 1:400 IF, #ab125045 Abcam |
| AQP5 | Rabbit, 1:500 IF, #A4985 Merck |
| AQP6 | Rabbit, 1:500 IF, #AQP61-A Alpha Diagnostic International |
| Beclin-1 | Mouse, 1:2000 WB, NBP1-00085 Novusbio |
| MAP-LC3-𝛽 | Mouse, 1:1000 WB, sc-271625 Santa Cruz Biotechnology |

**Suppl. Table 2:** primers used

| Primer | Forward sequence | Reverse sequence |
| --- | --- | --- |
| S18 | TCCCAGTAAGTGCGGGTCATA | CGAGGGCCTCACTAAACCATC |
| SOX2 | GCTAGTCTCCAAGCGACGAA | GCAAGAAGCCTCTCCTTGAA |
| OCT4 | TGTACTCCTCGGTCCCTTTC | TCCAGGTTTTCTTTCCTAGC |
| NANOG | CAGTCTGGACACTGGCTGAA | CTCGCTGATTAGGCTCCAAC |
| CD44 | ACACACGAAAGCAGGA | CACTGGGGTGGAATGTGTCT |
| CDH1 | CAGGTCTCCTCTTGGCTCTG | GACCGGTGCAATCTTCAAAA |
| αSMA | CCTGAAGAGCATCCCACCCT | ACCATCTCCAGAGTCCAGCACG |
| SNAIL | CCATTTCTGTGGAGGGAGGG | CCAGTGAGTCTGTCAGCCTTTGT |
| SPC | CTGGTTACCACTGCCACCTT | TCAAGACTGGGGATGCTCTC |
| AQP3 | CCTGGTGATGTTTGGCTGTGGCTC | TTCAGGTGGGCCCCAGAGACC |
| AQP5 | GGTGGTGGAGCTGATTCTGA | GAAGTAGATTCCGACAAGGTGG |
| AQP6 | Hs_AQP6_1_SG QuantiTect Primer Assay QT00010633, Qiagen | |
| Beclin-1 | CAGCCGAAGACTGAAGGTCA | TGCATTCCTCACAGAGTGGG |
| LC3 | CAGCATCCAACCAAAATCCCG | GTTGACATGGTCAGGTACAAGGA |
